# Soma size and Ca_v_1.3 channel expression in vulnerable and resistant motoneuron populations of the SOD1^G93A^ mouse model of ALS

**DOI:** 10.14814/phy2.12113

**Published:** 2014-08-08

**Authors:** Liza Shoenfeld, Ruth E. Westenbroek, Erika Fisher, Katharina A. Quinlan, Vicki M. Tysseling, Randall K. Powers, Charles J. Heckman, Marc D. Binder

**Affiliations:** 1 Graduate Program in Neurobiology & Behavior, University of Washington, Seattle, Washington, USA; 2 Department of Pharmacology, University of Washington School of Medicine, Seattle, Washington, USA; 3 Department of Physiology & Biophysics, University of Washington School of Medicine, Seattle, Washington, USA; 4 Department of Physiology, Northwestern University Feinberg School of Medicine, Chicago, Illinois, USA; 5 Department of Physical Therapy and Human Movement Sciences, Northwestern University Feinberg School of Medicine, Chicago, Illinois, USA; 6 Department of Physical Medicine and Rehabilitation, Northwestern University Feinberg School of Medicine, Chicago, Illinois, USA

**Keywords:** ALS, Ca_v_1.3 channels, motoneuron size, motoneurons in SOD1^G93A^ mice

## Abstract

Although the loss of motoneurons is an undisputed feature of amyotrophic lateral sclerosis (ALS) in man and in its animal models (SOD1 mutant mice), how the disease affects the size and excitability of motoneurons prior to their degeneration is not well understood. This study was designed to test the hypothesis that motoneurons in mutant SOD1^G93A^ mice exhibit an enlargement of soma size (i.e., cross‐sectional area) and an increase in Ca_v_1.3 channel expression at postnatal day 30, well before the manifestation of physiological symptoms that typically occur at p90 (Chiu et al. 1995). We made measurements of spinal and hypoglossal motoneurons vulnerable to degeneration, as well as motoneurons in the oculomotor nucleus that are resistant to degeneration. Overall, we found that the somata of motoneurons in male SOD1^G93A^ mutants were larger than those in wild‐type transgenic males. When females were included in the two groups, significance was lost. Expression levels of the Ca_v_1.3 channels were not differentiated by genotype, sex, or any interaction of the two. These results raise the intriguing possibility of an interaction between male sex steroid hormones and the SOD1 mutation in the etiopathogenesis of ALS.

## Abstract (precis)

This study was designed to test the hypothesis that motoneurons in mutant SOD1G93A mice exhibit an enlargement of soma size (i.e., cross‐sectional area) and an increase in Cav1.3 channel expression at postnatal day 30, well before the manifestation of physiological symptoms that typically occur at p90 (Chiu et al. 1995). We made measurements of spinal and hypoglossal motoneurons vulnerable to degeneration, as well as motoneurons in the oculomotor nucleus that are resistant to degeneration. Overall, we found that the somata of motoneurons in male SOD1G93A mutants were larger than those in wild‐type transgenic males. When females were included in the two groups, significance was lost. These results raise the intriguing possibility of an interaction between male sex steroid hormones and the SOD1 mutation in the etiopathogenesis of ALS.

## Introduction

Some of the first morphological evaluations of motoneurons in amyotrophic lateral sclerosis (ALS) were performed at or near end stage of the disease. Kiernan and Hudson (1991, 1993) reported a decrease in number and cross‐sectional area of motoneurons in both the spinal cord and the hypoglossal nucleus of deceased ALS patients. The authors contend that this observation can be explained by either a selective loss of large motoneurons, or by a disease‐induced shrinking of motoneurons. Similarly, mice with a rapidly progressing variant of the SOD1 mutation (SOD1^G86R^) showed significantly reduced neuronal volume and number in the facial nucleus, and trends towards reduced size and number in the hypoglossal nucleus at end stage (Nimchinsky et al. 2000).

At early postnatal stages, however, multiple morphological and electrophysiological reports suggest that vulnerable motoneurons are abnormally enlarged. Reconstructions of spinal lumbar motoneurons at p4‐9 in a slow progressing mSOD1 model (SOD1 ^G85R^) reveal an overall increase in neuron size in mutant SOD1 mice as compared to their wild‐type littermates. Here, the increase is driven by expansion of the dendritic tree, as no changes were evident in soma size (Amendola and Durand 2008). Lumbar motoneurons from p6‐10 in two lines of mutant SOD1 mice (SOD1 ^G86R^ and SOD1 ^G93A^) also showed a decreased input resistance and increased capacitance, indicative of larger size (Bories et al. 2007; Quinlan et al. 2011).

These two lines of evidence are not necessarily in conflict. Mutated SOD1 protein may instigate excessive motoneuron growth as part of a suite of early postnatal pathological processes. Compensatory mechanisms may prolong the survival of these enlarged neurons, until a point is reached at which compensation is overwhelmed by the disease processes. Evidence suggests that motoneuron death then proceeds in order from largest to smallest (Frey et al. 2000; Pun et al. 2006), allowing the survival of only small motoneurons at end stage. The course of these progressive changes highlights the importance of evaluating abnormalities at early age points in addition to end stage.

A number of additional abnormalities in SOD1 mutant mice can be detected before symptom onset, including the following: increased neuronal excitability, impaired axon transport, alterations in glutamate receptors, deficits in mitochondrial function, protein ubiquitination, and an imbalance in calcium handling (reviewed in Ling et al. 2013; Redler and Dokholyan 2012; Rothstein 2009). Of these, calcium dynamics may provide a window into ALS pathology, as motoneuron populations resistant to the disease show atypically high calcium buffering capacity (Vanselow and Keller 2000). Calcium enters neurons through either ligand‐gated channels or voltage gated channels. The voltage‐gated L‐type calcium channel (Ca_v_1.3) is integral to homeostasis as it mediates the calcium component of persistent inward currents (CaPIC) and can also open to admit calcium at rest (Xu and Lipscombe 2001). Intracellularly, calcium can be buffered by low levels of the calcium‐binding proteins calbindin and parvalbumin (if present), but in the vulnerable motoneurons that lack these proteins, calcium is largely taken up by mitochondria (Lautenschläger et al. 2013). As a result, extensive mitochondrial transport to dendritic space is required to maintain calcium homeostasis. Dysfunction at any of these steps could result in elevated intracellular calcium, which serves as a trigger for a number of degenerative cascades, including protein aggregation, mitochondrial dysfunction, and induction of proapoptotic pathways (reviewed in Choi 1988).

One of the earliest (p6‐12) detectable changes in SOD1 mutant motoneurons is an increase in the CaPIC (Quinlan et al. 2011) that serves to amplify synaptic inputs, facilitates repetitive firing and may endow motoneurons with bistable firing patterns (rev in Powers and Binder 2001). In the mouse, the Ca_v_1.3 channel mediating this current develops from little expression at birth to full adult expression levels at two to three weeks of age. The amplitude of the CaPIC increases concurrently (Jiang et al. 1999). Whereas both wild‐type and mutant SOD1 mice show elevations in CaPIC amplitude during postnatal development, the change is significantly more pronounced in motoneurons of the mutant SOD1 mouse. This is likely a result of either accelerated neuronal maturation, or overexpression of the Ca_v_1.3 calcium channel that underlies the CaPIC (Quinlan et al. 2011).

Interestingly, the early postnatal enlargement of motoneuron size and the increase in CaPIC may in fact be linked. As the disease induces a pathological enlargement in motoneuron size, excitability should decrease. To obviate reduced excitability, however, neurons may up‐regulate expression of the Ca_v_1.3 channel responsible for mediating the CaPIC, a current integral in establishing the excitability of motoneurons (Powers and Binder 2001). Supporting this hypothesis, in early postnatal mutant SOD1 motoneurons input conductance was found to increase along with CaPIC amplitude (Quinlan et al. 2011), yielding an essentially unaltered net excitability. These findings suggest motoneurons may utilize CaPIC enhancement as a compensatory mechanism to maintain excitability despite size increases.

To explore whether there is indeed a correlation between increases in motoneuron size and the upregulation of Ca_v_1.3 channel expression, this study employed a mutant SOD1^G93A^ mouse (Gurney et al. 1994), the most widely used animal model for ALS. This mouse model expresses a human mutant version of the SOD1 gene with a glycine to alanine substitution at position 93, and develops pathology mimicking ALS in humans. Clinical disease onset is marked at 90 days, and by the time mice reach end stage at p136, spinal motoneuron pools show losses of nearly 50% (Chiu et al. 1995). To evaluate changes in size and calcium channel expression in adult presymptomatic mice, motoneurons were evaluated at p30, at which point they do not yet show overt degeneration (Hegedus et al. 2007), but do show alterations in input conductance (Elbasiouny et al. 2010). A transgenic mouse expressing the wild‐type variant of the SOD1 gene was used as a control group to account for any possible alterations brought on by increased protein expression (Tortarolo et al. 2004).

To compare the effects of the SOD1 mutation on vulnerable and resistant motoneuron populations, we evaluated vulnerable motoneurons of the hypoglossal nucleus, cervical, and lumbar spinal cord (Gurney et al. 1994), as well as resistant motoneurons of the oculomotor nucleus (Nimchinsky et al. 2000; Haenggeli and Kato 2002). All three populations (spinal, hypoglossal, and oculomotor) show extensive expression of the Ca_v_1.3 channel (Sukiasyan et al. 2009), permitting analysis of how the SOD1 mutation may differentially affect Ca_v_1.3 channel density in the three motoneuron groups.

We anticipated that staining for the Ca_v_1.3 channel would be denser in motoneurons of SOD1 mice, particularly in the vulnerable hypoglossal and spinal populations. Given that CaPIC amplitude is increased by p12 in this model, it was reasonable to expect a notable increase in channel expression at p30. In addition, this study tested the hypothesis that an increase in expression levels of Ca_v_1.3 which gives rise to the CaPIC is a compensatory reaction to increased motoneuron size. Accordingly, it was expected that vulnerable motoneurons would be larger in the mutant SOD1 mouse. We were also interested in the confound of sex on the different motoneuron pools, as several reports have indicated that male humans and male mutant mice are more susceptible to the disease progression than females (Veldink et al. 2003; Suzuki et al. 2007; McCombe and Henderson 2010).

Overall, we found no significant difference between soma area of both male and female motoneurons from mice transfected with the SOD1^G93A^ mutant gene and its wild‐type counterpart (SOD1^WT^). We did find, however, that the somata of motoneurons in male SOD1^G93A^ mutants were larger than those in wild‐type transgenic males. Expression levels of the Ca_v_1.3 channels were not differentiated by genotype, sex, or any interaction of the two. These results raise the intriguing possibility of an interaction between male sex steroid hormones and the SOD1 mutation in the etiopathogenesis of ALS.

## Materials and Methods

### Animals

We studied perfused brain tissue of p29–p31 transgenic mice overexpressing the human SOD1^G93A^ gene, the human SOD1^WT^ gene, nontransgenic, and/or expressing GFP driven by the Hb9 promoter. Data were collected from 25 mice raised at the animal facilities at Northwestern University: nine nontransgenic (five female, four male), eight SOD1^WT^ (four female, four male), and eight SOD1^G93A^ (four female, four male). The brains were coded at Northwestern University prior to shipment to Seattle to ensure an unbiased, blinded analysis at the W.M. Keck Microscopy Facility at the University of Washington. The SOD1^G93A^ and SOD1^WT^ genes were identified using standard PCR techniques (Rosen et al. 1993). Briefly, 20–25 mg of tissue was used for the DNA extraction. The primers for amplification are SOD1: CAG TAA CTG AGA GTT TAC CCT TTG GT (forward) and CAC ACT AAT GCT CTG GGA AGA AAG A (reverse) and Hb9:eGFP:AAG TTC ATC TGC ACC ACC G. All mice were used according to Northwestern University's Animal Care and Use Committee guidelines.

### Materials

Avidin, biotin, and biotinylated goat anti‐rabbit IgG were purchased from Vector (Burlingame, CA). Streptavidin 555 was purchased from Invitrogen (Life Technologies, Grand Island, NY).

Antibodies (anti‐CND1) that specifically recognize the *α*1 subunits of class D (Ca_V_1.3) Ca^2+^ channels were used in this study. Their generation, purification, and characterization have been reported previously (Hell et al. 1993). Briefly, the CND1 peptide (KYDNKVTIDDYQEEAEDKD; residues 809 to 825, Hui et al. 1991) corresponds to highly variable sites within the intracellular loops between domain II and III of class D *α*1 subunits of rat brain calcium channels. The NH2‐terminal lysine and tyrosine were added for cross‐linking and labeling purposes and are not part of the channel sequences. Peptides were synthesized by the solid‐phase method (Merrifield 1963), purified by reverse‐phase HPLC on a Vydac 218 TP10 column, and confirmed by amino acid analysis. The purified peptides were coupled with glutaraldehyde to bovine serum albumin (Orth 1979), dialyzed against PBS, and emulsified in the same volume of Freund's complete (initial injection) or incomplete adjuvant. Injections were done in multiple subcutaneous sites on New Zealand white rabbits at three week intervals. Antisera were collected, and antibodies were purified by affinity chromatography on CND1 peptides coupled to CNBr‐activated Sepharose. Two mL of antiserum were adsorbed to the column matrix and incubated at room temperature for 5 h with stirring on a tilting mixer. The columns were then washed with TBS, and bound IgG was eluted with 0.1 mol/L glycine (pH 2.7). The affinity‐purified anti‐CND1 was brought to neutral pH using 0.1 mol/L Tris. (Hell et al. 1993)

### Surgery

Mice were euthanized with C0_2_, and trans‐cardially perfused with 4% paraformaldehyde in phosphate buffer (PB). The central nervous system was carefully dissected out and submerged in tubes of 4% paraformaldehyde in PB overnight at 4°C. The tissue was then cryoprotected by sinking them in 30% sucrose in PB at 4°C until it was processed for histology.

### Immunocytochemistry

Tissue sections (40 *μ*m) were cut on a sliding microtome, placed in 0.1 M phosphate buffer, and then processed for immunocytochemistry as described previously (Westenbroek et al. 1998). Briefly, tissue sections were rinsed in 0.1 mol/L Tris buffer (TB) pH 7.4 for 15 min, in 0.1 mol/L Tris buffered saline (TBS) for 15 min, blocked using 2% avidin in TBS for 30 min., rinsed in TBS for 30 min., blocked in 2% biotin in TBS for 30 min., and finally rinsed in 0.1M TBS for 30 min. The tissue sections were then incubated in affinity‐purified anti‐CND1 (diluted 1:50) for 36 h at 4°C. All antibodies were diluted in a solution containing 0.1% Triton X‐100 and 1% NGS in 0.1M TBS. The tissue sections were rinsed in TBS for 60 min and incubated in biotinylated goat anti‐rabbit IgG diluted 1:300 for 1 h at 37°C. The tissue was then rinsed in TBS for 30 min, incubated in Streptavidin 555 diluted 1:750 for 1 hr at 37°C, then rinsed with TBS for 10 min., rinsed with TB for 20 min and mounted on charged microscope slides (Fisherbrand Superfrost/Plus), and coverslipped with AquaMount (Fisher).

### Image acquisition and analysis

Gain‐ and offset‐matched images were collected on a Leica SL confocal microscope in the W.M. Keck Microscopy Facility at the University of Washington. All z‐stacks were collected in 1 *μ*m thick sections. To determine the level of nonspecific staining, sections were incubated without primary antibody and the immunocytochemical reaction was then carried out as described above. Sections stained in the absence of primary antibody showed no detectable labeling. A threshold function was applied to all z‐stack images, which were hand traced using a Bamboo Create digitizing tablet interfaced with Fiji Image J. Motoneuron size and staining density measurements were taken using the Region of Interest (ROI) function in Fiji Image J, which compiled traced outlines of the neuron soma. Each soma was measured for cross‐sectional area, mean stain intensity (8‐bit, max intensity), and roundness, a measure of circularity ranging from 0 to 1 (perfect circle). Roundness measurements were generated using the fit ellipse function on Fiji Image J and selecting “shape descriptors” in the measurement options panel.

### Neuron selection

In the spinal cord, slices were obtained from all available cervical and lumbar sections. Prominent neurons in the lateral ventral horn were selected and traced for each of the spinal cord sections. The hypoglossal nucleus was clearly delineated by the Ca_v_1.3 stain, and all visible neurons were selected within the boundaries of the nucleus. The oculomotor nucleus was identified with the aid of a comparative Nissl/Ca_v_1.3 staining atlas, and all neurons within boundaries of the nucleus were selected for analysis. Following established guidelines, for all nuclei a subset of the neurons traced were selected as motoneurons based on a threshold of diameter ≥ 20 um (Sukiasyan et al. 2009).

### Statistical analysis

Data regarding cross‐sectional soma area, roundness, and stain intensity were first pooled across the three genotypic groups (nontransgenic, SOD1^wt^, SOD1^G93A^) using a one‐way ANOVA test for significance. The ANOVA model, however, does not take into account the correlation that occurs in observations from the same mouse. Observations from the same mouse cannot be treated as independent, as there may be underlying characteristics not captured in the ANOVA model that contribute to similarities within a single mouse.

To obviate this concern about statistical validity, consultation was sought from a statistics advisory group at the University of Washington (Rachael Maltiel, Bailey Fosdick, Brittany Sanchez, and Paul Sampson). They developed a “mixed linear effects model” to account for both fixed and random effects (Kutner et al. 2003). To implement this statistical model, the genetic groups were narrowed down to the two of interest: the mutant SOD1^G93A^ group and the SOD1^wt^ group, which serves as the statistical control.

The following describes how the linear mixed effects model generates “expected” values. For soma cross‐sectional area, we set Area_*ij*_ as the size of the *j*th observation from mouse *i*. All the explanatory variables are categorical 0‐1 indicators of nucleus region, genotype, and sex that a particular observation Area_*ij*_ pertains to. The reference categories are as follows: oculomotor (nucleus region), SOD1^wt^ (genotype), and female (sex), meaning that the intercept term β_0_ is the fitted average area of an oculomotor neuron from a SOD1^wt^ female. The model is expressed as follows:


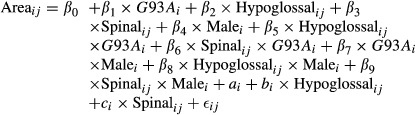



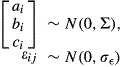


The model also includes a random effect *a*_*i*_ for mouse *I*, capturing mouse‐to‐mouse variability for the reference region group. The random slope effects *b*_*i*_*, c*_*i*_ capture mouse‐to‐mouse variability of differences from oculomotor to hypoglossal and from oculomotor to spinal. *ε*_ij_ represents the variability evident within a single mouse and region across multiple observations.

A custom script written in the *R* software programming language was used to generate fitted model coefficients and their corresponding significance values for each of the fixed and random effects, for the parameters of area, roundness, and stain. For the area analysis, the model described above was transformed with a natural logarithmic function to generate more evenly distributed residuals in a QQ plot assessing normality of errors. No transformation was necessary in assessing roundness and stain intensity.

## Results

**Figure 1.**
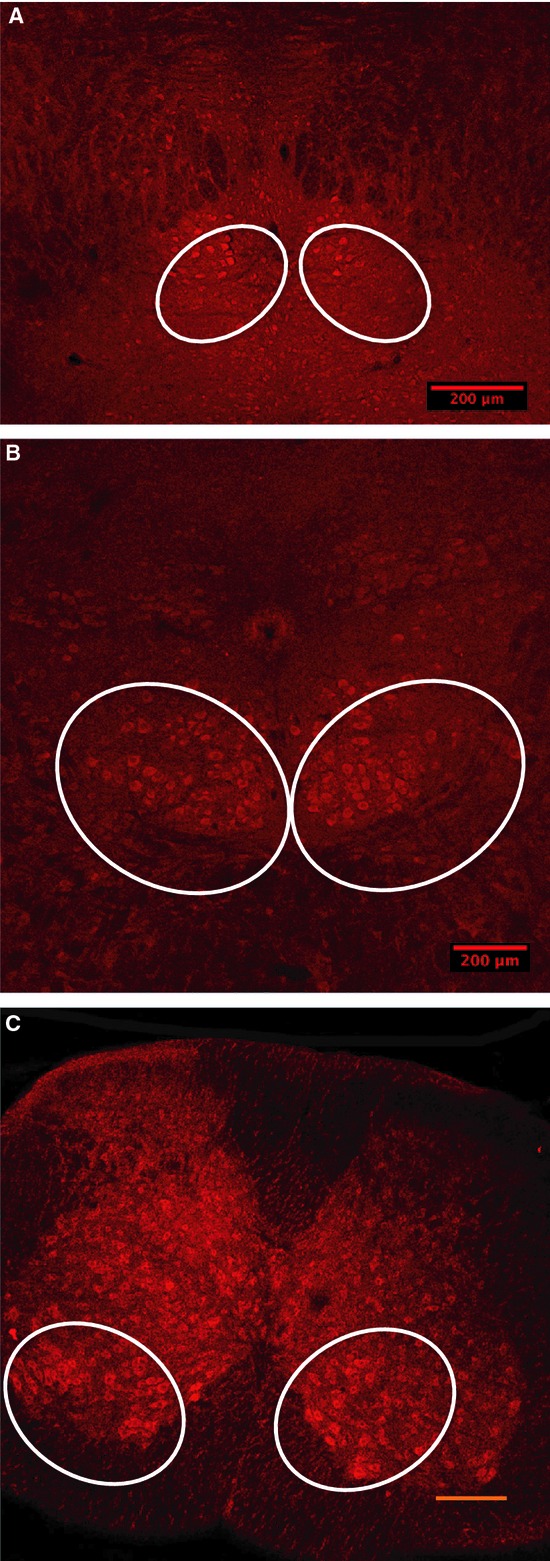
Representative confocal images of Ca_v_1.3 staining in the oculomotor (A), hypoglossal (B), and spinal (C) nuclei. Oval traces indicate region boundaries used for selecting motoneurons. Staining for Ca_v_1.3 is intense and widespread, but particularly concentrated in the large somata of motoneurons.

**Figure 2.**
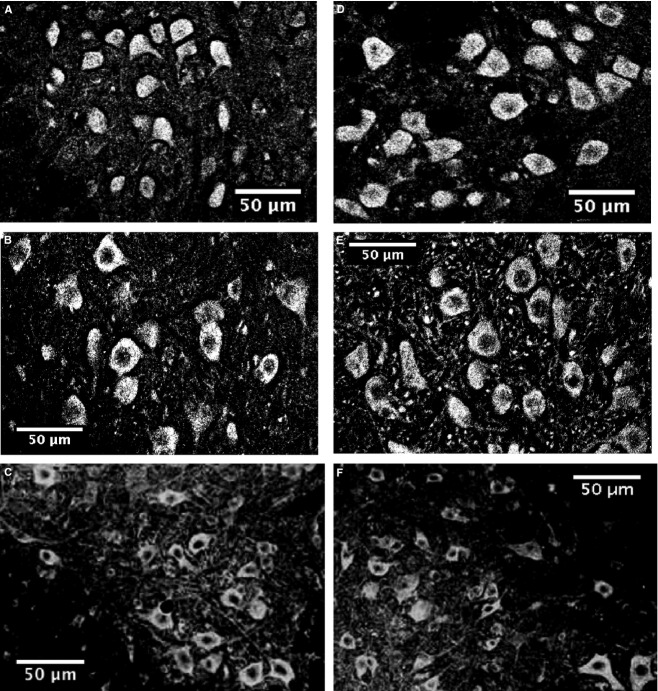
Representative confocal images of Ca_v_1.3 staining in SOD1^WT^ (A–C) and SOD1^G93A^ (D–F) mice in the oculomotor nucleus (A and D), hypoglossal nucleus (B and E), and spinal cord (C and F). Images were acquired through a 20x lens, and threshold‐adjusted on black and white spectrum on Fiji Image J for optimal visualization of soma size and shape. Ca_v_1.3 staining is intense throughout the soma in all regions and genotypes analyzed.

The immunocytochemical staining for Ca_v_1.3 channels revealed broad and intense expression of the channel throughout the oculomotor, hypoglossal, and spinal nuclei (Fig. 1). Expression levels are so consistently high that the Ca_v_1.3 stain was used not only to measure channel density, but also to serve as a neuronal marker for the measurements of soma size and shape. Confocal images taken with a higher magnification (20×) showed that the stain was clear and bright throughout nuclei, with no obvious qualitative differences in Ca_v_1.3 expression levels between SOD1^wt^ and SOD1^G93A^ mice, in any of the three regions analyzed (Fig. 2).

**Figure 3.**
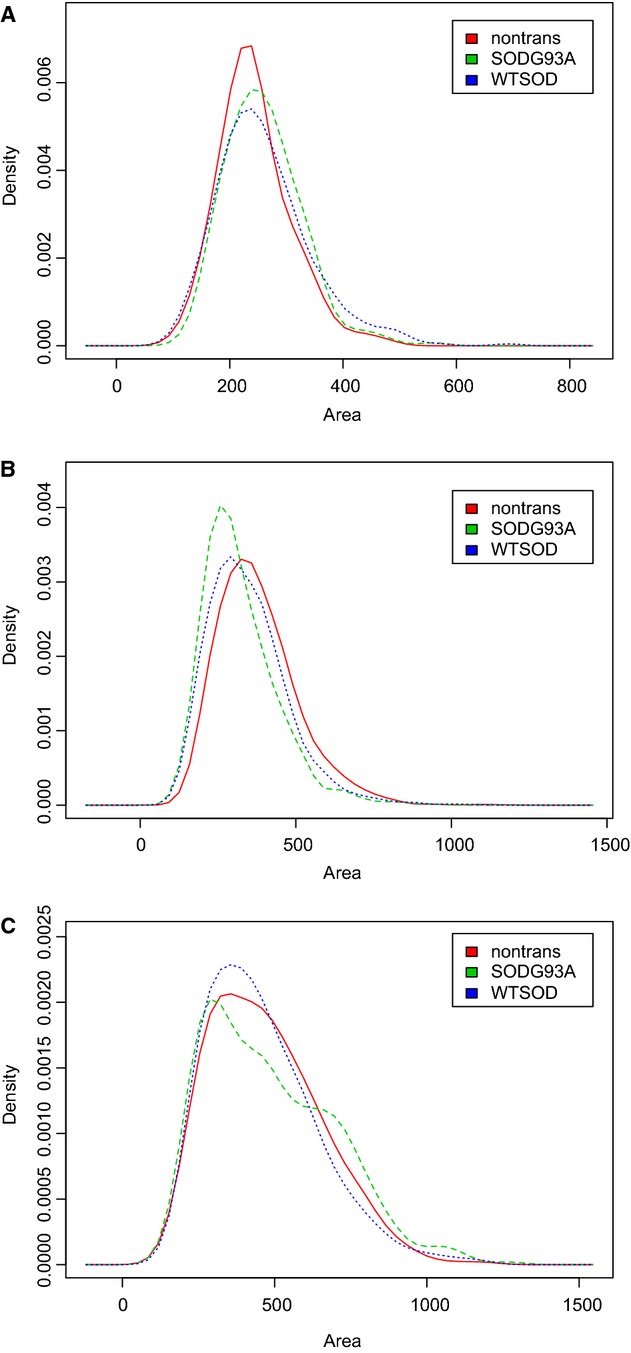
Density plots of somatic size of motoneurons in oculomotor (A), hypoglossal (B), and spinal (C) nuclei sorted by genotype: nontransgenic, SOD1^G93A^, and SOD1^WT^. Due to the statistical power afforded by an ANOVA with large sample sizes (>2000 neurons per group), small but statistically significant differences were revealed between groups for all three nuclei regions. However, the order of the size differences failed to show a consistent pattern across the three regions.

We measured the soma sizes of spinal (*n* = 1738; from 19 animals), oculomotor (*n* = 1140; from 15 animals) and hypoglossal motoneurons (*n* = 6596; from 25 animals) and initially analyzed the data using a standard one‐way ANOVA. The ANOVA revealed small, but statistically significant differences between the three genotypic groups (SOD1^G93A^, SOD1^wt^, and nontransgenic) for all three nuclei regions (Fig. 3). The order of the size differences, however, failed to show a consistent pattern.

In the oculomotor nucleus, motoneurons of SOD1^wt^ mice (mean = 260 µm^2^, SD = 83; 358 motoneurons from 5 animals) and SOD1^G93A^ mice (mean = 259 µm^2^, SD = 67; 524 motoneurons from seven animals) were indistinguishable, whereas motoneurons in nontransgenic mice were smaller (mean = 244 µm^2^, SD = 63; 258 motoneurons from three animals). A one‐way ANOVA revealed significant differences between the groups (*P* = 0.015), and Tukey post hoc tests showed that the SOD1^G93A^ and SOD1^wt^ groups were significantly larger than the nontransgenic group (*P* = 0.04 and 0.02, respectively), but not different from each other.

In the hypoglossal nucleus, motoneurons of nontransgenic mice were the largest (m = 378 µm^2^, SD = 129; 3239 motoneurons from nine animals), followed by SOD1^wt^ (m = 345 µm^2^, SD = 127; 1899 motoneurons from eight animals), with SOD1^G93A^ motoneurons (m = 320 µm^2^, SD = 117; 1458 motoneurons from eight animals) showing the smallest average area (*P* ≪ 0.001 in a one‐way ANOVA). Post hoc Tukey tests also yielded significant differences in each group comparison (*P *≪ 0.001).

Data from spinal motoneurons provide an additional inconsistency. Motoneurons of SOD1^G93A^ mice were the largest (m = 489 µm^2^, SD = 211; 360 motoneurons from five animals), followed by nontransgenic motoneurons (m = 471 µm^2^, SD = 179; 701 motoneurons from six animals), and by SOD1^wt^ motoneurons (m = 458 µm^2^, SD = 178; 677 motoneurons from eight animals). A one‐way ANOVA showed the difference in group means to be significant (*P* = 0.03), and Tukey post hoc tests showed that this difference stems from the comparison between the SOD1^wt^ and SOD1^G93A^ groups (*P* = 0.03); all other comparisons revealed no significant differences in soma size.

**Figure 4.**
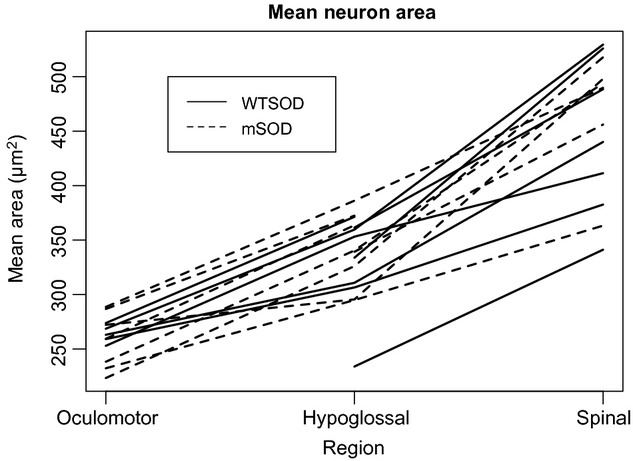
Average observed soma cross‐sectional area by region across mice distinguished by genotype group (SOD1^wt^ and mutant SOD1^G93A^). Lines connect the average areas across regions in a single mouse to show individual differences in addition to regional and genotypic differences. Soma sizes are generally largest for spinal motoneurons, followed by motoneurons of the hypoglossal nucleus, and smallest for oculomotor neurons. Appreciable variation is evident between individual mice. Also of note, there does not appear to be a clear distinction between soma sizes pertaining to mice of SOD1^wt^ versus SOD1^G93A^ genotype.

The use of a one‐way ANOVA presents a number of problems for the present analyses. Ideally, statistical analyses should parse out effects generated by genotype, sex, and region analyzed, as well as interactions between each of those factors. Thus, expanding the model to a two‐way ANOVA would still be ineffective in analyzing three converging factors. More importantly, the ANOVA fails to account for individual differences within genotype groups. A valid statistical model cannot treat different observations from the same mouse as independent, as individual variation may confound the analysis. Indeed, Figure 4 shows that average observed soma cross‐sectional area varies significantly from mouse to mouse. As a consequence, motoneuron measurements from different animals in our study could not be pooled.

To capture individual variability, and also to incorporate data from all animal groups and regions analyzed, a linear mixed effects model was employed as described in the Methods section. As the model is best suited to a comparison of two groups, the analysis focused on mice of SOD1^G93A^ and SOD1^wt^ genotype. SOD1^wt^ mice serve as a more rigorous control group than the nontransgenic mice, as simply transfecting mice with a human gene, whether or not that gene predisposes the animal to disease, may alter morphology and physiology (Efiok and Safer 2000; Tortarolo et al. 2004).

With individual differences taken into account, the present sample sizes drop substantially (Table 1). When each neuron was counted as a sampling unit, total sample size exceeded 5000. When observations from a single mouse were pooled together, the sample size totaled just 16, split evenly among the sex and genotype combination groups. This results in a sizable decrease in statistical power, but does lend greater validity to the analysis.

**Table 1.** Sample sizes for sex and genotype groupings counted by number of neurons traced (total = 5276) and by number of animals evaluated per grouping (total = 16). When each neuron is counted as a single observation, the sample sizes in each group are quite large. However, when data are collapsed across animals to account for individual differences, the sample size is reduced substantially. Using the number of animals as a sample size rather than the number of neurons reduces the statistical power but gives greater statistical validity to the analysis

	*N*, neurons	*N*, animals
Male, SOD1^G93A^	1112	4
Female, SOD1^G93A^	1230	4
Male, SOD1^wt^	1292	4
Female, SOD1^wt^	1642	4
Total	5276	16

**Figure 5.**
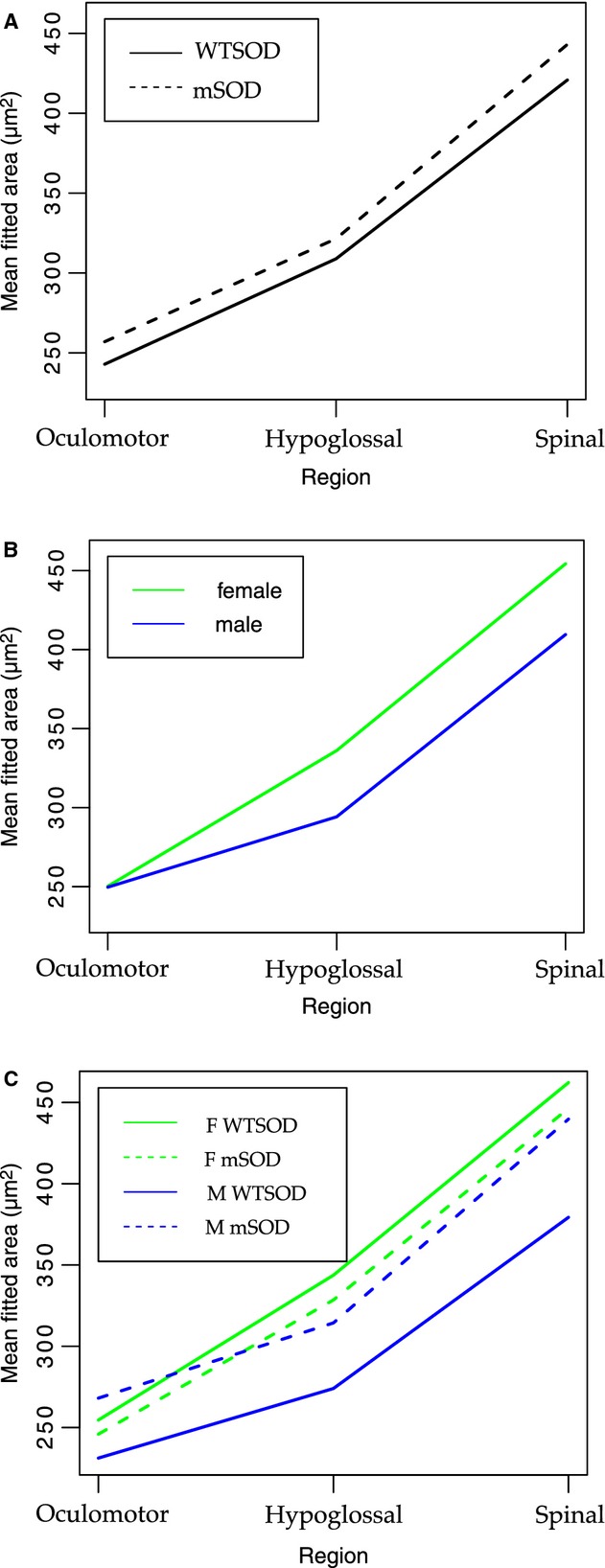
Fitted average soma cross‐sectional area for oculomotor, hypoglossal, and spinal motoneurons when collapsed across sex to give comparison by genotype (A), collapsed across genotype to give comparison by sex (B), and separated by sex and genotype to show group specific trends (C). Fitted average areas were calculated according to the mixed linear effects model, which was also used to calculate effect size and statistical significance of variable coefficients pertaining to genotype, sex, and region. Although it appears in (A) that soma sizes are slightly larger in SOD1^G93A^ than SOD1^wt^ for all regions, the model did not find this difference to be significant. Nor did the model find an overall sex difference in soma area, although as evident in (B), there is an interaction effect between the hypoglossal region and male sex, leading to significantly smaller fitted area. A nonparallel relationship between SOD1^G93A^ and SOD1^wt^ status for females and males is evident in (C), indicating that the mutant genotype and male sex interact to enlarge soma area (see Table ****2 for model coefficients and statistical significance).

Fitted average soma cross‐sectional areas, as calculated by the mixed effects model, are shown in Figure 5, with corresponding coefficients and statistical results listed in Table 2. Overall, there was no effect of genotype (SOD1^G93A^ vs. SOD1^WT^) when other factors (sex and motoneuron region) were held constant. Nor was there an effect of sex when genotype and motoneuron region were held constant. However, the model did reveal that male hypoglossal motoneurons were significantly smaller than female hypoglossal motoneurons. Also significant was the size order, from spinal motoneurons (largest) to hypoglossal to oculomotor (smallest), all other factors held constant. The most striking finding is that the motoneuron somata of male SOD1^G93A^ mutants are larger than their wild‐type counterparts in all three motor regions (Fig. 5C).

**Table 2.** Fitted model coefficients for linear mixed effects model giving soma area. The model uses a reference group of oculomotor neurons from a SOD1^WT^ female; all values listed here are generated in comparison with this reference group. For soma area, the model was log transformed to normalize residuals. All significant coefficients are in bold and include the following: intercept (as expected; a nonsignificant intercept would indicate that the mean log area of the reference group is 0), hypoglossal and spinal groups (indicating greater soma area in these groups than the reference oculomotor group), and an interaction between the hypoglossal group and male sex (indicating a nonparallel relationship between SOD1^G93A^ status and sex)

	Estimate	*P*‐value
**β**_**0**_ **=Intercept**	**5.54**	**<0.001**
β_1_=SOD1^G93A^	−0.03	0.583
**β**_**2**_ **=Hypoglossal**	**0.30**	**<0.001**
**β**_**3**_ **=Spinal**	**0.60**	**<0.001**
β_4_=Male	−0.10	0.17
β_5_=Hypoglossal × SOD1^G93A^	−0.01	0.752
β_6_=Spinal × SOD1^G93A^	0	0.997
β_7_=SOD1^G93A ^× Male	0.18	0.062
**β**_**8**_ **=Hypoglossal × Male**	**−0.13**	**0.002**
β_9_=Spinal × Male	−0.10	0.136

**Figure 6.**
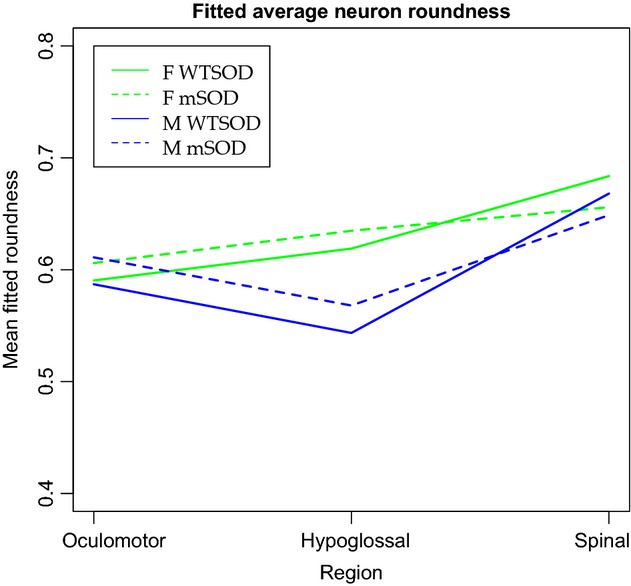
Mean fitted roundness values for oculomotor, hypoglossal, and spinal motoneurons grouped by sex and genotype. Roundness was determined by the ratio of major diameter to minor diameter in a best‐fit ellipse generated by ImageJ, and ranges from 0 to 1 (perfect circle). There are no overall statistically significant differences in roundness between genotypes or sexes, although the model does reveal rounder motoneurons in the spinal cord, and an interaction effect between the hypoglossal region and the male sex leading to less round motoneurons.

To analyze motoneuron shape in addition to size, roundness values were calculated for each motoneuron based on the ratio between the major and minor diameters of a best‐fit ellipse (Bernard et al. 2012). The model did not reveal any overall difference in motoneuron roundness by genotype or by sex (Fig. 6). All other factors fixed, spinal motoneurons are significantly rounder than hypoglossal or oculomotor motoneurons (*P* < 0.05), and an interaction effect between the hypoglossal region and the male sex led to significantly less round motoneurons (*P* < 0.05).

**Figure 7.**
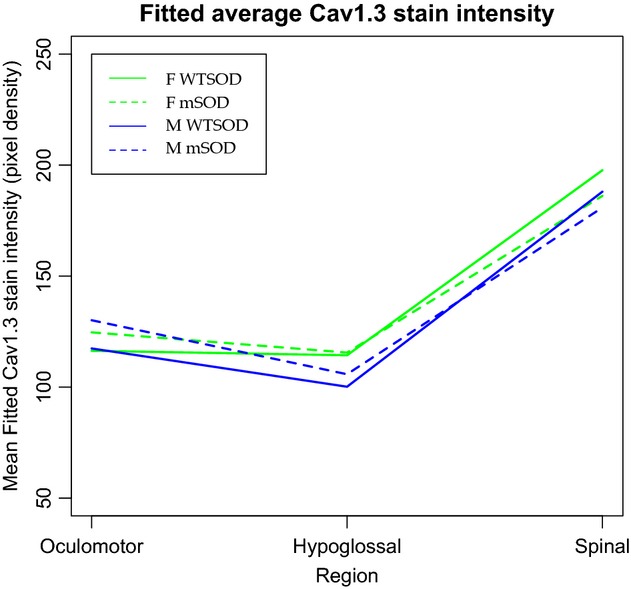
Mean fitted Ca_v_1.3 stain intensity for oculomotor, hypoglossal, and spinal motoneurons grouped by sex and genotype. Stain intensity was measured by the average pixel density of grouped z‐stacks in Fiji ImageJ, and ranges from 0 to 256. The model used to analyze stain density incorporated an additional random effect for date of image acquisition, as laser intensity on the confocal microscope can vary day‐to‐day. There are no overall statistically significant differences in Ca_v_1.3 staining intensity between genotypes or sexes. The model did confirm more intense staining in motoneurons of the spinal cord than of the hypoglossal or oculomotor nuclei.

Analyzing the intensity of the stain for the Ca_v_1.3 channel presented additional complications. Because the strength of the confocal laser can vary by day, the model was adjusted to include an effect for imaging date. Including this random effect removed the variability associated with changes in the confocal laser, but did make differences more difficult to parse out. Overall, staining for Ca_v_1.3 is brighter in the spinal motoneurons and does not appear to show any other consistent pattern (Fig. 7). The model did not detect any differences due to genotype, sex, or any interaction therein, but did confirm brighter staining in the spinal cord than in the hypoglossal or oculomotor nuclei (*P* < 0.01).

## Discussion

This study was designed to test the hypothesis that motoneurons in mutant SOD1^G93A^ mice exhibit an enlargement of soma size (i.e., cross‐sectional area) and an increase in Ca_v_1.3 channel expression at p30, well before the manifestation of physiological symptoms that typically occur at p90 (Chiu et al. 1995). We made measurements of spinal and hypoglossal motoneurons vulnerable to the mutation, as well as motoneurons in the oculomotor nucleus that are resistant. Overall, we only found significant differences in soma size between male SOD1^G93A^ mutant and SOD1^WT^ motoneurons. When females were included with males in the SOD1^G93A^ and SOD1^WT^ groups, the significance in soma size was lost. Our analyses indicated no significant differences are present in motoneuron shape, as measured by the roundness of the soma. Similarly, levels of the Ca_v_1.3 channel staining were not differentiated by genotype, sex or any interaction of the two.

These results find mixed support in a conflicting body of literature regarding the effects of the SOD1^G93A^ mutation on early postnatal motoneuron size. A number of studies report an increase in total input conductance, an electrical measurement indicative of larger neuronal size, in early postnatal SOD1^G85R^ and SOD1^G93A^ motoneurons (Bories et al. 2007; Elbasiouny et al. 2010; Quinlan et al. 2011), although no difference was reported in p4‐10 hypoglossal motoneurons (van Zundert et al. 2008). However, morphological analyses show no difference in soma size between SOD1^G93A^ and SOD1^WT^ motoneurons (Kuo et al. 2004; Amendola and Durand 2008).

The most parsimonious explanation for these seemingly disparate results may be that motoneuron enlargement is rooted in an expansion of the dendritic tree, which will affect physiological measurements, and can occur independently of any increase in soma size (Amendola and Durand 2008). Indeed, Amendola and Durand (2008) report a proliferation of distal dendritic branches in lumbar motoneurons of young SOD1 mice, without any change in soma size, number, or mean diameter of primary dendrites. This study, which was limited to somatic dimensions as a metric for motoneuron size, may have failed to capture real differences that exist between the mutant and wild‐type animals.

The one striking difference we did observe was an interaction effect between sex and SOD1^G93A^ genotype: namely that the motoneurons in mutant SOD1 males are significantly larger than those in wild‐type SOD1 males. This finding raises two questions: does the incidence and course of ALS‐like symptoms in SOD1^G93A^ mice differ between males and females? And further, how might the mutant SOD1^G93A^ protein interact differently with motoneurons by sex?

A recent literature review of sex differences in ALS concluded that the incidence and prevalence of ALS are greater in men than in women. However, this sex difference is found only in large studies including both sporadic and familial ALS cases, and disappears when familial ALS is studied independently (McCombe and Henderson 2010). Given that the SOD1 mouse is thought to model the familial form of the disease, it is logical to question whether the animal model adequately captures the sex differences found in the sporadic form, which accounts for 90–95% of cases. In fact, SOD1 mutant mice do show earlier disease onset in males than in females (Veldink et al. 2003; Suzuki et al. 2007), although no effect of sex on loss of muscle force is evident (Hegedus et al. 2009).

What is it about being male that might accelerate the onset of the disease, and can the root be traced to differences in the physiology of motoneurons? Sex differences in disease course typically raise the possibility of the involvement of sex steroid hormones in etiopathogenesis. In ALS, there is limited but intriguing evidence for hormone involvement. The male sex is associated with higher levels of prenatal testosterone (Slob et al. 1980). However, a recent examination of finger length ratios, a surrogate marker for prenatal testosterone levels, revealed that both men and women with ALS were exposed to higher prenatal testosterone than were healthy controls (Vivekananda et al. 2011).

In embryonic cultures, motoneurons transfected with human androgen receptors exhibit a dose‐dependent change in morphology in response to androgen treatment, developing larger cell bodies and broader dendritic processes (Brooks et al. 1998). Thus, elevated prenatal testosterone levels may predispose motoneurons to abnormal growth. Further, this predisposition may be more likely to actualize in males, whose naturally higher testosterone levels may be more precariously balanced between its beneficial effects on motoneuron survival (Jones 1994), and induction of excessive growth (Brooks et al. 1998).

Very little is known about whether sex steroid hormones may also influence expression levels of the Ca_v_1.3 calcium channel. In the only known study of this possible relationship, Ca_v_1.3 mRNA levels were found to be higher in male wild‐type mice than in females (Li et al. 2010). The present data did not uncover any sex differences in expression levels of Cav1.3. Nor were any differences detected between mutant SOD1^G93A^ and SOD1^WT^ genotypes. These result appears to stand in contrast with a report of greater CaPIC amplitude in juvenile mutant SOD1^G93A^ mice (Quinlan et al. 2011).

There are several possible explanations that may reconcile the enhanced CaPIC amplitude in mutant SOD1^G93A^ mice mentioned above (Quinlan et al. 2011) with the absence of an evident change in somatic expression of the Ca_v_1.3 channel responsible for that current reported here. The CaPIC is largely a current of dendritic origin (Lee and Heckman 1996; Carlin et al. 2000). The Ca_v_1.3 channel is expressed widely throughout the neuron body and its processes (Sukiasyan et al. 2009), but modulation of its expression may be localized to its more active presence in dendritic processes. Ca_v_1.3 expression may not change at the soma in the SOD1^G93A^ mouse, but may be up‐regulated in the dendritic tree, which could not be imaged using the techniques employed in this study.

Alternatively, it is possible that the CaPIC is elevated not through an expansion of the number of Ca_v_1.3 channels, but through an increase in the open probability or activation threshold of the existing channel population. van Zundert et al. (2008) reported an increase in early postnatal NaPIC without a corresponding increase in the density of the responsible channels. The authors suggest that the effect is achieved instead through a premature Na channel isoform turnover to isoforms that permit greater levels of persistent activity. Recently, several splice variants of the Ca_v_1.3 channel have been identified, composing a range of activation thresholds and open probabilities (Bock et al. 2011). Whereas the effects of the SOD1^G93A^ mutation on Ca_v_1.3 isoform diversity have yet to be explored, modulation of channel variant presents one possible pathway to overall current enhancement.

Technical limitations of Ca_v_1.3 immunocytochemistry and confocal microscopy meant that only the motoneuron somata could be analyzed for channel expression and size. Since both neuron growth (Amendola and Durand 2008) and CaPIC (Carlin et al. 2000) have been shown to be highly concentrated in the dendritic regions, future studies should employ techniques that allow for data acquisition from the dendritic tree. In addition, our measurements of immunostaining include an unknown fraction of nonfunctional cytostolic channels en route to the membrane. Finally, future studies should examine mutant SOD1^G93A^ effects at a range of age points. The timeline of ALS pathology has proved difficult to parse out; a better understanding of temporal relationships between pathological events will help distinguish between primary and compensatory mechanisms (Quinlan 2011).

## Conflict of Interest

None declared.
